# Metabolite-Specific Echo Planar Imaging for Preclinical Studies with Hyperpolarized ^13^C-Pyruvate MRI

**DOI:** 10.3390/tomography9020059

**Published:** 2023-03-27

**Authors:** Sule I. Sahin, Xiao Ji, Shubhangi Agarwal, Avantika Sinha, Ivina Mali, Jeremy W. Gordon, Mark Mattingly, Sukumar Subramaniam, John Kurhanewicz, Peder E. Z. Larson, Renuka Sriram

**Affiliations:** 1 UC Berkeley—UCSF Graduate Program in Bioengineering, Berkeley, CA 94720, USA; 2 Department of Radiology and Biomedical Imaging, University of California, San Francisco, CA 94016, USA; 3 Bruker, Billerica, MA 01821, USA

**Keywords:** echo planar imaging (EPI), chemical shift imaging (CSI), hyperpolarized ^13^C magnetic resonance imaging (MRI)

## Abstract

Metabolite-specific echo-planar imaging (EPI) sequences with spectral–spatial (spsp) excitation are commonly used in clinical hyperpolarized [1-^13^C]pyruvate studies because of their speed, efficiency, and flexibility. In contrast, preclinical systems typically rely on slower spectroscopic methods, such as chemical shift imaging (CSI). In this study, a 2D spspEPI sequence was developed for use on a preclinical 3T Bruker system and tested on in vivo mice experiments with patient-derived xenograft renal cell carcinoma (RCC) or prostate cancer tissues implanted in the kidney or liver. Compared to spspEPI sequences, CSI were found to have a broader point spread function via simulations and exhibited signal bleeding between vasculature and tumors in vivo. Parameters for the spspEPI sequence were optimized using simulations and verified with in vivo data. The expected lactate SNR and pharmacokinetic modeling accuracy increased with lower pyruvate flip angles (less than 15°), intermediate lactate flip angles (25° to 40°), and temporal resolution of 3 s. Overall SNR was also higher with coarser spatial resolution (4 mm isotropic vs. 2 mm isotropic). Pharmacokinetic modelling used to fit k_PL_ maps showed results consistent with the previous literature and across different sequences and tumor xenografts. This work describes and justifies the pulse design and parameter choices for preclinical spspEPI hyperpolarized ^13^C-pyruvate studies and shows superior image quality to CSI.

## 1. Introduction

Magnetic resonance imaging (MRI) with hyperpolarized [1-^13^C]pyruvate, using the dissolution dynamic nuclear-polarization technique, can interrogate key crossroads of metabolism by measuring whether pyruvate is converted to lactate, alanine, or enters the TCA cycle. Elevated pyruvate to lactate conversion is a hallmark of many cancers, known as the Warburg effect, motivating the use of this modality for cancer imaging. Hyperpolarized ^13^C-pyruvate MRI is now in clinical trials at 13 institutions worldwide with applications including prostate cancer [1], brain tumors [2], breast cancer [3], kidney cancer [4], kidney disease, liver disease, ischemic heart disease, and cardiomyopathies [5,6].

Metabolite-specific imaging is a popular tool in clinical hyperpolarized [1-^13^C]pyruvate MRI as it provides excellent performance in terms of speed, coverage, and acquisition flexibility compared to spectroscopic imaging and chemical shift-encoding methods [7,8,9]. Specifically, this technique uses a spectrally and spatially selective (spsp) excitation pulse followed by a fast imaging readout, such as echo planar imaging (EPI). With a typical spspEPI sequence, one metabolite is excited at a time, and for each metabolite, all of k-space is acquired within one TR. Other metabolite-specific sequences have also been used previously, such as balanced steady-state free precession (bSSFP), which allowed for improved SNR by refocusing transverse magnetization [10,11]. Metabolite-specific imaging has become the optimal choice for hyperpolarized [1-^13^C]pyruvate studies of tumor metabolism due to the sparsity of spectral information of interest.

Recent optimizations at our institute for clinical spspEPI include mitigation of B_0_ inhomogeneities by improved shimming using proton signals [12], avoidance of flip angle variation over time due to its sensitivity to transmit B_1_ inhomogeneities, and calibration errors [13], and incorporation of real-time calibrations of hyperpolarized agent bolus arrival time, B_0_, and B_1_ to improve the reproducibility and SNR for better quantification [14].

In contrast, the standard for hyperpolarized [1-^13^C]pyruvate MRI experiments on preclinical imaging systems is a chemical shift imaging (CSI) or non-localized spectroscopic sequences. While this sequence is very robust and requires no knowledge of the chemical shifts, it is also much slower than spspEPI. Fast spectroscopic imaging approaches, such as EPSI, have been developed as an alternative to CSI [15]. However, EPSI sequences still fall short of metabolite-specific sequences, such as spspEPI, in terms of speed and flexibility [8].

There has been limited development of spspEPI ^13^C sequences on preclinical imaging systems and scarcely any implementation in preclinical in vivo research studies [16,17]. Thus, the objective of this work is to reverse engineer a 2D metabolite-specific clinical EPI protocol to a preclinical imaging system. In this, we include comparisons to CSI sequences, and additionally analyze different acquisition parameters as well as perform rate constant fitting for metabolite-specific EPI in murine studies.

## 2. Materials and Methods

Simulations: Simulations of the hyperpolarized signals were performed to evaluate the expected spatial resolution, SNR, and pharmacokinetic model accuracy for the EPI and CSI sequences. Magnetization decay across the k-space was simulated for both EPI and CSI acquisitions, taking into account RF effects, T_1_ decay, and metabolic conversion. The metabolism was simulated using a pharmacokinetic model with one physical compartment and two chemical pools—pyruvate and lactate [18]. The decay across one TR, from TR = *n* − 1 to *n*, can be modeled as:

(1) $$M_{n}=\mathrm{rot}\left(\alpha \right)\cdot \mathrm{spoil}\cdot \mathrm{expm}\left(R\cdot \mathrm{TR}\right)\cdot M_{n-1}$$

where *M* stores the transverse and longitudinal magnetizations of pyruvate and lactate, i.e., $M=\left[M_{P,x},M_{P,y},M_{P,z},M_{L,x},M_{L,y},M_{L,z}\right]{}^{\prime }$, and expm is a matrix exponential. The matrix R includes relaxation and metabolic conversion and can be defined as:

(2) $$R=\left\{\begin{array}{cccccc} 1 & 0 & 0 & 0 & 0 & 0\\ 0 & 1 & 0 & 0 & 0 & 0\\ 0 & 0 & -k_{PL}^{\prime \prime }-R_{1P} & 0 & 0 & 0\\ 0 & 0 & 0 & 1 & 0 & 0\\ 0 & 0 & 0 & 0 & 1 & 0\\ 0 & 0 & k_{PL}^{\prime \prime } & 0 & 0 & -R_{1L} \end{array}\right\}$$

where *k_PL_*″ is the effective apparent rate constant and R_1*P*_ and R_1*L*_ are the reciprocals of pyruvate and lactate T_1_. The matrix rot is a 3D rotation matrix to simulate RF excitation and spoil is a matrix that eliminates transverse magnetization (M_x_ and M_y_) after each TR.

The model used parameters of *k_PL_* = 0.05 1/s, T_1,pyruvate_ = 20 s, and T_1,lactate_ = 30 s with values chosen based on prior in vivo fitting results. A realistic input function was modelled as a gamma distribution with FWHM of 8 s determined from the previous in vivo datasets. The input function was used to simulate bolus effects and inflow of injected pyruvate.

The point spread function (PSF) was simulated by taking the IFFT of the magnetization decay across k-space. The full width at half maximum (FWHM) was calculated for a y = 0 slice of the PSF, including zero-filling k-space before the IFFT. T_2_* blurring effects were not considered due to short EPI readout time (100 ms) and expected relatively long T_2_ of metabolites [19].

The pharmacokinetic model was also used to simulate average pyruvate and lactate dynamic curves for EPI. Equation (1) can be modified and expanded to multiple time points for an EPI sequence. For time point t, first, pyruvate is acquired:

(3) $$\begin{array}{c} M_{t,P}={\mathrm{rot}}_{P}\left(\alpha_{P}\right)\cdot {\mathrm{spoil}}_{P}\cdot \left(M_{t-1}+U_{t}\right)\\ P_{t}=M_{t,P}^{\left(1\right)}+iM_{t,P}^{\left(2\right)} \end{array}$$

Here, *U_t_* stores the input function for time point t and is added to longitudinal pyruvate magnetization. The matrix rot_P_ is a rotation matrix that only rotates pyruvate magnetizations (simulating the spsp RF pulse) and *P_t_* is the complex pyruvate signal for timepoint *t*. Next, lactate is acquired:

(4) $$\begin{array}{c} M_{t,L}={\mathrm{rot}}_{L}\left(\alpha_{L}\right)\cdot {\mathrm{spoil}}_{L}\cdot \mathrm{expm}\left(R\cdot {\mathrm{TR}}_{P}\right)\cdot M_{t,P}\\ L_{t}=M_{t,L}^{\left(4\right)}+iM_{t,L}^{\left(5\right)} \end{array}$$

Similarly, *L_t_* is the complex lactate signal for timepoint *t*. Finally, relaxation and metabolic conversion at the delay at the end of each timepoint is modeled to arrive at the final magnetization vector, *M_t_*, at the end of timepoint *t*:

(5) $$M_{t}=\mathrm{expm}\left(R\cdot \left({\mathrm{TempRes}-\mathrm{TR}}_{P}\right)\right)\cdot M_{t,L}$$

This model was repeated for all timepoints to simulate complex pyruvate and lactate signal over time. The relative SNR was approximated as the sum of the modelled magnitude signal over time. A Monte Carlo simulation was performed using simulated dynamic curves with random noise to fit *k_PL_* values [18]. The standard deviation of the fit *k_PL_* values across Monte Carlo iterations was calculated as a measure of *k_PL_* fitting precision.

**Figure 1:**
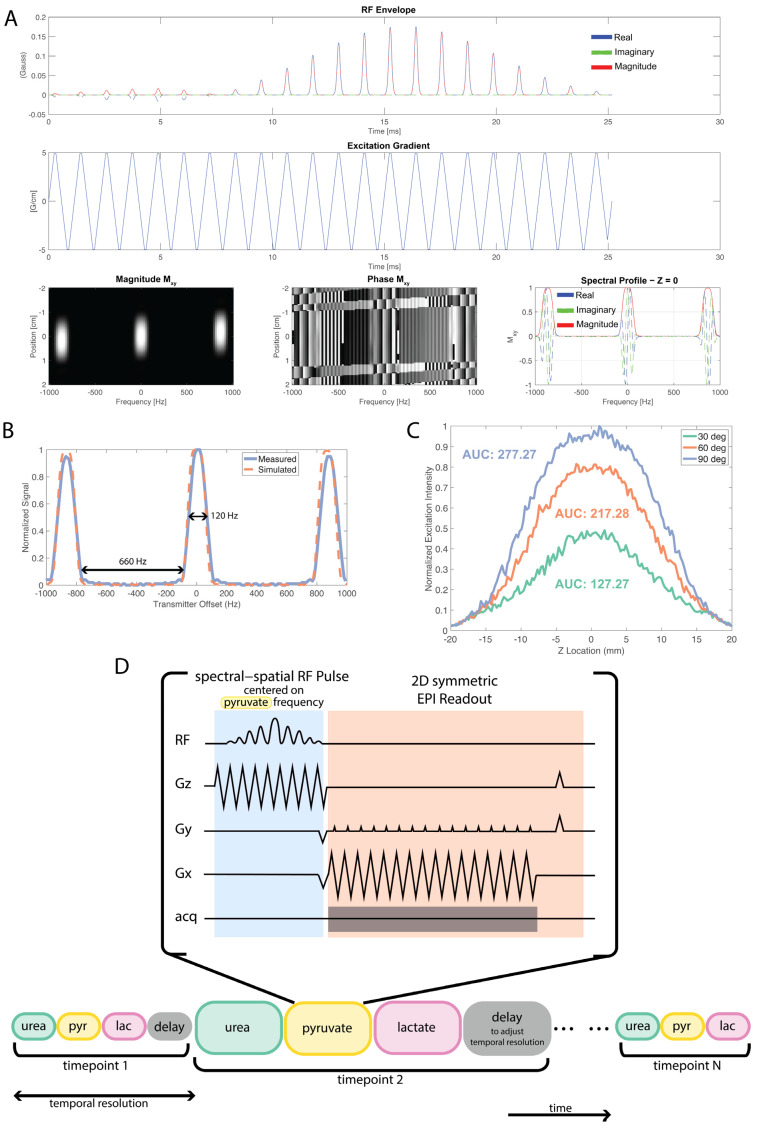
EPI spectral-spatial RF pulse design. (**A**) RF pulse design using the SPSP RF pulse design toolbox [20]. (**B**) SPSP RF pulse frequency selectivity measurements were performed with a 4M ^13^C Urea sphere phantom. For this pulse, the measured passband had a FWHM of 120 Hz and stopband of 660 Hz. (**C**) Slice profile and corresponding area-under-the-curve (AUC) values for 30°, 60°, and 90° excitations. Corresponding FWHM values are 16.77 mm, 18.68 mm, and 20.10 mm, respectively. (**D**) During one timepoint, each metabolite is acquired using a spsp RF pulse with an EPI readout. Delay time is added at the end of a timepoint to achieve preferred temporal resolution.

Pulse Sequences: A ramp-sampled, symmetric EPI readout with spsp excitation was developed for use on the ParaVision 6.0 Bruker software (Bruker, Billerica, MA, USA). The original EPI sequence on the Bruker scanner (Bruker, Billerica, MA, USA) was modified for ^13^C applications and for use with spsp pulses, by enabling multiple image acquisitions pertaining to different chemical shifts, with variable flip angle implementation. Dynamic images were acquired over a minute with 18 metabolite images for each chemical shift in the following sequence, urea, pyruvate, and lactate, every 3 or 4 s. A reference scan was used to correct for the EPI phase errors that lead to Nyquist ghosts. This also enabled the online reconstruction of ^13^C metabolite maps. The spsp RF pulse used in all of the EPI acquisitions was designed to individually excite [1-^13^C]pyruvate, [1-^13^C]lactate, or ^13^C Urea at 3T, with a passband FWHM of 120 Hz and a stopband of 600 Hz (Figure 1). The spectral spatial pulse was 25.17 ms long and designed with a default slice thickness of 15 mm and is the same pulse used in the clinical studies [7]. The RF pulse was designed in MATLAB (MathWorks, Natick, MA, USA) using the spsp RF pulse design toolbox [20]. Further information and access by request to the pulse sequence can be found through https://github.com/UCSF-HMTRC/ (accessed on 18 March 2023)

Experiments: All experiments were performed on a preclinical 3T cryogen-free Bruker Biospin (Billerica, MA, USA) with a maximum gradient strength of 960 mT/m and a maximum slew rate of 3550 T/m/s. A dual-tuned 40 mm ^1^H/^13^C volume coil was used for both the thermal phantom and hyperpolarized ^13^C experiments. A total of 24 mg of [1-^13^C]pyruvate was polarized on Hypersense (Oxford Instruments, Oxford, England). Thermal phantoms consisted of a ~2 cm diameter sphere filled with 4M ^13^C-Urea doped with 25mM Magnevist. A 5 mm diameter and 4 cm long cylindrical tube filled with 4M ^13^C urea was used with mice during in vivo testing for calibration.

Eight mice implanted with patient-derived xenograft tissues of either renal cell carcinoma (RCC), or prostate cancer (LTL610, LuCap93) in the kidney or liver were used for testing the sequences (RCC kidney: 3, LTL liver: 2, LTL kidney: 2, LuCap93 kidney: 1) [21,22,23,24]. Animal experiments were performed under protocols approved by the Institutional Animal Care and Use Committee. Mice were anesthetized during the MRI experiments and their tail veins were cannulated for infusion of hyperpolarized solutions. Then, 350 μL of hyperpolarized neutralized 80 mM [1-^13^C]pyruvate was infused in 12–15 s. Imaging was started 10 s after the start of the infusion. The average time between hyperpolarized imaging experiments was 41 min. For the 8 studies where both spspEPI and CSI imaging were acquired, CSI was acquired before spspEPI for 6 of them.

Different flip-angle combinations were tested for the metabolite-specific EPI sequence: 10° for urea, 10°, 15°, or 20° for pyruvate and 20°, 30°, or 50° for lactate. The field-of-view acquired was either 3.2 cm × 3.2 cm or 6.4 cm × 6.4 cm and corresponding spatial resolutions of 2 mm × 2 mm or 4 mm × 4 mm. Temporal resolutions tested were 3 or 4 s, adjusted by adding delay time after acquisition of metabolites (Figure 1D). Corresponding CSI scans were acquired with flip angle 10°, resolution 4 mm × 4 mm with FOV of 3.2 cm × 3.2 cm, centric phase encode ordering, and temporal resolution of 4.25 s. CSI flip angle was chosen due to previous success using flip angle (see Figure S1 for example dynamics). Multi-slice ^1^H T_2_ RARE images were acquired with a 3.2 cm × 3.2 cm FOV and 0.167 mm × 0.167 mm resolution for anatomical reference.

Analysis: CSI metabolite maps were generated using SIVIC [25]. Tumor regions-of-interest (ROIs) were drawn on T_2_ RARE images and down-sampled to the EPI and CSI resolutions. Hyperpolarized signal time courses were normalized by the mean noise over time within a region outside of the animal. An inputless one-physical-compartment pharmacokinetic model accounting for RF pulse flip angles was used to fit *k_PL_* to the average signal from all tumor voxels resulting in a single *k_PL_* value and per voxel within the tumor resulting in a *k_PL_* map [18,26]. Hyperpolarized images and *k_PL_* maps were overlaid on T_2_ RARE after resampling to the T_2_ RARE resolution and applying a Fermi filter. Simulations, models, and analysis were performed using MATLAB (Mathworks, Natick, MA, USA).

## 3. Results

### 3.1. Spectral–Spatial Pulse Design and Calibration

The spectral–spatial RF pulse design and pulse profile measurements are shown in Figure 1. There is excellent agreement between the simulated and measured frequency profile of the pulse, confirming the 120 Hz FWHM passband and a stopband of over 600 Hz. The spsp pulse power was calibrated on a 4M ^13^C-urea sphere phantom by using a one-dimensional slab acquisition and stepping through the power for a 23.5 ms pulse length and determining the 90° flip angle that corresponds to the maximal signal (when covering a full 180 nutation). Additionally, the varying RF flip angle and subsequent slice profile of the pulse was tested in the spspEPI sequence using a long 4M Urea phantom as demonstrated in Figure 1C. The FWHM of the slice profile for 30°, 60°, and 90° excitations were 16.77 mm, 18.68 mm, and 20.10 mm, respectively. The AUC values were 127.27, 217.28, and 277.27, respectively.

### 3.2. Comparison of EPI and CSI

#### 3.2.1. Simulations

**Figure 2:**
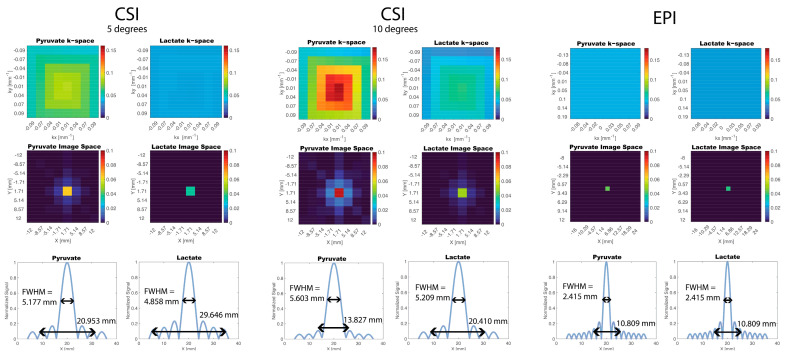
Simulation of PSF for CSI and EPI Acquisition. Magnetization decay was modelled across k-space using a pharmacokinetic model. The CSI PSFs resulted in signal blurring in image space whereas the EPI PSF resulted in a delta function. The CSI PSFs for two different flip angles are shown: 5 degrees and 10 degrees.

A 2D PSF was simulated for both CSI and EPI acquisitions (Figure 2). The simulated EPI PSF was effectively a delta function with no signal blurring across voxels whereas the simulated CSI PSF resulted in signal blurring for both pyruvate and lactate due to varying signal amplitudes across phase encoding steps. From the CSI PSF profile with a 10 degree flip angle, the FWHM was 5.603 mm and 5.209 mm for pyruvate and lactate, respectively, which is broader than the nominal resolution of 4 mm. The width at 10% of max signal was 13.827 mm and 20.410 mm for pyruvate and lactate. In comparison, for the EPI PSF profile, the FWHM and width at 10% of max signal was 2.415 mm and 10.809 mm for both pyruvate and lactate. For in vivo experiments, the simulations indicate that CSI will have a higher likelihood of signal bleeding, for example between the vasculature to kidney tumors, compared to EPI. This would typically result in lower *k_PL_* estimates for voxels adjacent to large vessels because of the additional vascular pyruvate signal bleed into nearby regions.

#### 3.2.2. In Vivo Experiments

**Figure 3:**
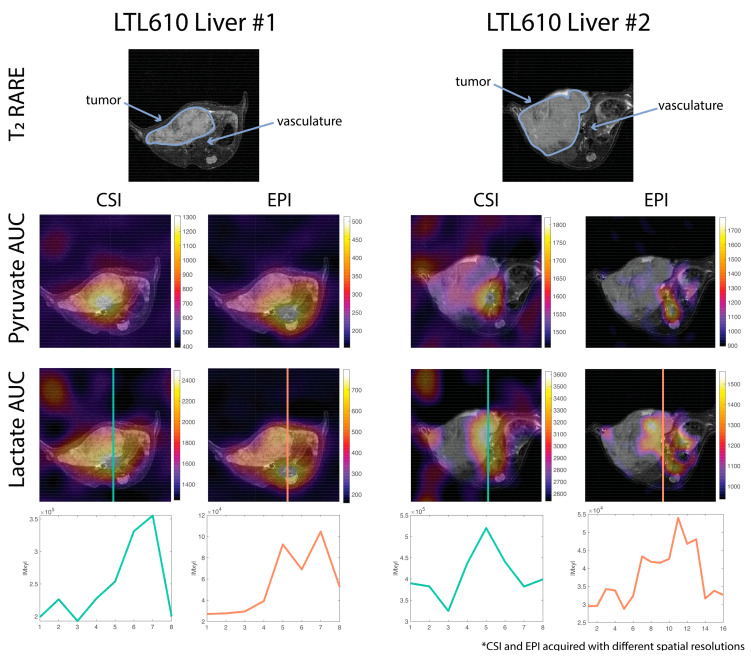
In vivo pyruvate and lactate area-under-the-curve (AUC) maps of two mice overlaid on T_2_ RARE images. Both examples are of an LTL610 tumor implanted in the mouse liver. The EPI maps and lactate line profiles show two distinct signal peaks whereas the CSI maps have a single peak. The blurring of the vasculature signal due to the CSI PSF obscures lactate signal from the tumor. For both examples, CSI was acquired before EPI. See Figure S1 for dynamics of the CSI acquisition.

Pyruvate and lactate AUC images of CSI and EPI scans overlaid on proton T_2_ images of the same mouse were compared side by side (Figure 3). CSI maps showed a strong metabolic signal from the vasculature but did not show any signal from the tumor region that was clearly differentiable from the vasculature signal. Meanwhile, the EPI maps showed metabolite signals that were better delineated and clearly originating from the tumor as well as the vasculature. Line plot profiles of the lactate signal show two distinct peaks for EPI compared to a single peak for CSI. This agrees with the PSF results from the simulation and suggests the tumor signal for the CSI maps is obscured by the blurred vasculature signal.

### 3.3. Testing In Vivo EPI Parameters Optimization for Robust SNR and k_PL_

#### 3.3.1. Simulations

**Figure 4:**
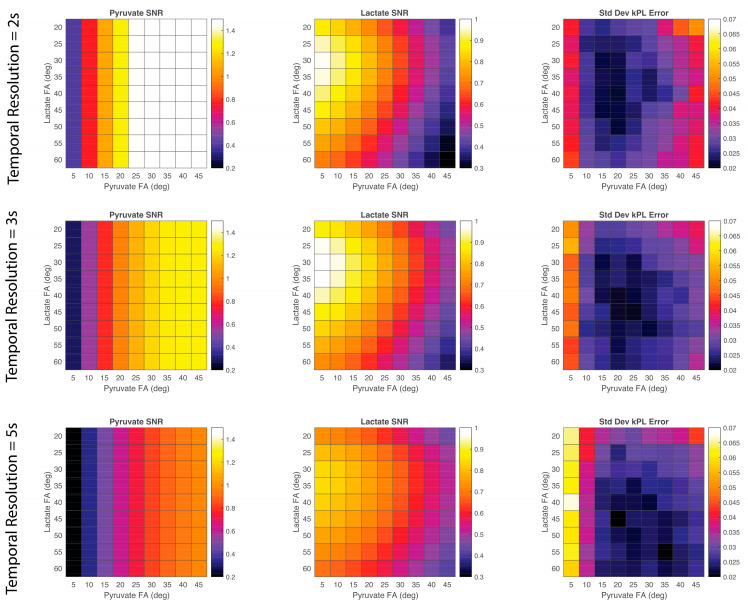
Pyruvate and lactate SNR and *k_PL_* fitting error of simulated EPI signal for various temporal resolutions and pyruvate and lactate flip angles (FAs). The simulation used the following parameters: *k_PL_* = 0.05 1/s; T_1,pyruvate_ = 20 s; and T_1,lactate_ = 30 s.

Relative pyruvate and lactate SNR was calculated for various pyruvate–lactate flip angle pairs and temporal resolutions that can be used in a metabolite-specific EPI experiments (Figure 4). Pyruvate SNR was only dependent on the flip angle of pyruvate and not lactate, as expected. Pyruvate SNR was the highest for pyruvate flip angles of 30 to 45 degrees. The highest lactate SNR resulted when using lower pyruvate flip angles (less than 15 degrees) and lactate flip angles between 25 and 40 degrees. For pyruvate–lactate flip angle pairs, the standard deviation of *k_PL_* fitting error was also calculated. For a temporal resolution of 2 s, *k_PL_* fitting error variation was the lowest for pyruvate flip angles of 15–20 degrees and lactate flip angles of 30 to 50 degrees. For a temporal resolution of 3 s, *k_PL_* fitting error variation was minimized for pyruvate flip angles of 15–25 degrees and lactate flip angles of 30–60 degrees. For a temporal resolution of 5 s, *k_PL_* fitting error variation was minimized for pyruvate flip angles of 20–35 degrees and lactate flip angles of 40–60 degrees.

A simulated EPI acquisition, with a shorter temporal resolution of 2 s, resulted in a relative increase in pyruvate SNR. In contrast, lactate SNR peaked with a temporal resolution of 3 s. A longer temporal resolution decreased the *k_PL_* fitting error for a mid-range of flip angles. According to the simulation, a pyruvate flip angle between 10° to 20°, a lactate flip angle of 25° to 40°, and a temporal resolution of 3 s will optimize lactate SNR without compromising pyruvate SNR and maintain a low *k_PL_* fitting error.

#### 3.3.2. In Vivo Experiments

The results of the simulation (Figure 4) were used to guide EPI parameter choices. A pyruvate flip angle of 15° and a lactate flip angle of 30° were chosen as the simulation suggested high lactate SNR and low *k_PL_* error. A smaller pyruvate flip angle was not used as previous experiments found shorter pyruvate flip angles did not provide sufficient signal. Larger flip angles of 20° and 50° for pyruvate and lactate were chosen for comparison. A temporal resolution of 3 s was chosen as lactate SNR was the highest and a 4 s temporal resolution was used for comparison. The feasibility of these chosen parameters was evaluated with in vivo experiments.

**Figure 5:**
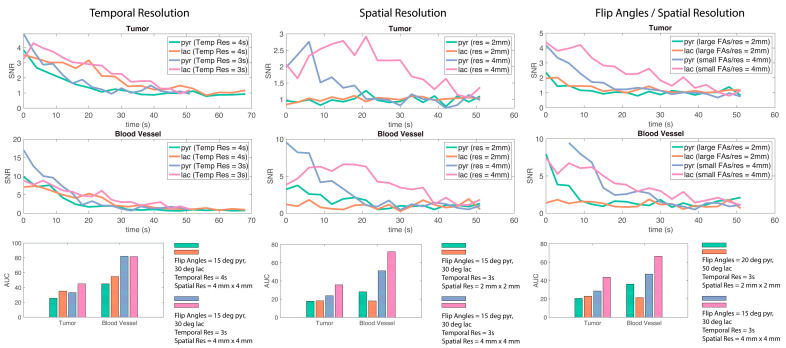
SNR over time and area-under-the-curve (AUC) values for various in vivo mice experiments. Shorter temporal resolution and coarser spatial resolution result in higher signal AUC. Tumor model and location from left to right: LuCap93 kidney; LTL kidney; and LTL kidney.

Average pyruvate and lactate time courses within tumor and blood vessel ROIs for EPI scans with varying parameters were plotted (Figure 5). Using a temporal resolution of 3 s resulted in higher relative SNR and area-under-the-curve (AUC) for pyruvate and lactate compared with a temporal resolution of 4 s. A coarser spatial resolution of 4 mm × 4 mm also resulted in a higher AUC for pyruvate and lactate compared with a finer spatial resolution of 2 mm × 2 mm. Additionally, a coarser spatial resolution paired with an optimized flip angle choice of 15° for pyruvate and 30° for lactate increased the AUC for pyruvate and lactate in the tumor compared with the finer spatial resolution and flip angles of 20° and 50° for pyruvate and lactate. These experiments showed that changing spatial resolution from 2 mm to 4 mm and temporal resolution from 4 s to 3 s improved pyruvate and lactate SNR. Nevertheless, it is important to consider that these comparisons were conducted with a very limited sample size and some of the data have limited SNR (Figure 5, center top) which interfere with drawing strong conclusions. Instead, these results support previously shown simulations (Figure 4).

**Figure 6:**
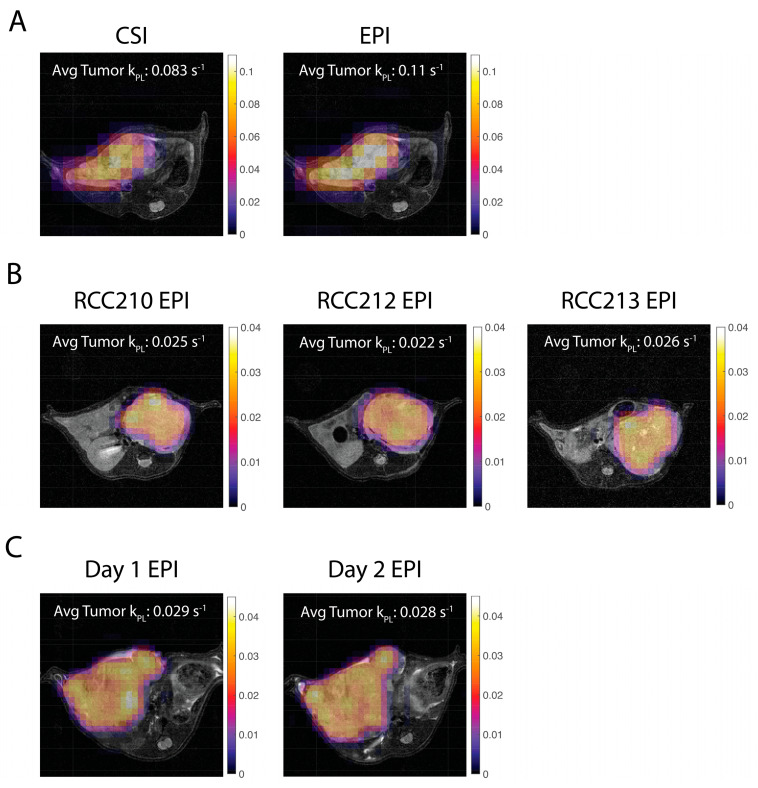
Pharmacokinetic model fits of *k_PL_* for in vivo mice overlaid on ^1^H T2 RARE images. (**A**) CSI and EPI *k_PL_* maps are consistent for the same mouse with an LTL liver tumor. (**B**) EPI *k_PL_* maps are consistent for three different mice with RCC tumors. (**C**) EPI *k_PL_* maps are consistent for the same mouse with an LTL liver tumor imaged on two consecutive days. All *k_PL_* values are as expected and similar to a previous in vivo study [27].

Voxel-wise *k_PL_* maps were calculated for CSI and EPI data in implanted tumors (Figure 6). In one mouse, the *k_PL_* values within the liver tumor were very similar between CSI and EPI sequences, and ranged from 0.08–0.11 1/s. In another mouse, EPI *k_PL_* values were consistent between two consecutive days for the same liver tumor with average tumor *k_PL_* of 0.029 1/s and 0.028 1/s. The three mice with the same type of implanted RCC tumors also demonstrated relatively similar *k_PL_* values of 0.02–0.035 1/s across the tumors, with some differences in heterogeneity between the animals. All *k_PL_* values were similar to the previous literature [27].

## 4. Discussion

We have successfully implemented and optimized a preclinical two-dimensional (2D) spsp EPI sequence to better match the state-of-the-art clinical hyperpolarized ^13^C metabolic imaging studies. These are being performed as part of a co-clinical trial of metastatic prostate cancer patients prior to and after chemotherapy which is a project funded under the Co-Clinical Imaging Research Program (U24CA253377).

### 4.1. Spectral–Spatial Pulse

We have presented testing procedures and results of RF power calibration and spatial selectivity that can be unique to the vendor implementation of the spsp pulse and its implementation in a sequence. Both parameters are critical for the spsp pulse performance.

### 4.2. CSI vs. spspEPI

A metabolite-specific EPI sequence with a spectral–spatial pulse can improve flexibility and innovation in preclinical hyperpolarized ^13^C studies. In this work, we demonstrate how a preclinical spspEPI pipeline may work as well as highlighting some of the advantages in comparison to CSI methods.

An advantage of a spspEPI sequence is its speed which can allow for shorter temporal resolutions for multi-metabolite studies, multi-slice, or even 3D acquisitions without losing spatial resolution. In addition, using a spspEPI acquisition may make translation of preclinical studies to the clinic straightforward in terms of expectation of signal observation, dynamics, and kinetic rates.

SpspEPI sequences also allow for flexibility in acquisition trajectories and flip angles. With a spsp pulse, the flip angle of an individual metabolite can be adjusted allowing for manipulation of the signal to improve the SNR of downstream metabolites [28]. The EPI trajectory supports variable spatial resolutions for an individual metabolite which can also be used to manipulate the signal to improve SNR [29].

Another consideration is signal blurring in CSI due to T_1_ decay and metabolic conversion experienced between signals acquired across phase encodings. This may be minimized with center-out k-space trajectories, but still leads to a broadening of the PSF especially compared to EPI. This was confirmed in this work with a simulation (Figure 2) and in vivo data (Figure 3). This blurring is particularly impactful when imaging small animals where the vasculature is anatomically close to regions or organs of interest. The higher vasculature signal may bleed into the signal from other organs making them difficult to differentiate and more difficult to quantify the metabolism. In our experience, a spspEPI acquisition allowed for better localization of signal in small animals.

### 4.3. Optimization of EPI Parameters

A spspEPI acquisition allows for more flexibility which in turn specifies more parameter choices. In this work, three parameters were optimized through simulations and confirmed with in vivo experiments. With a fast EPI readout, there is flexibility in the choice of temporal resolution dependent on the delay between time point acquisitions. Simulations demonstrated shorter temporal resolutions resulted in higher pyruvate SNR as expected since more of the hyperpolarized signal is captured before T_1_ decay. In contrast, lactate SNR peaks at a temporal resolution of 3 s as there is more time for metabolic conversion from pyruvate to lactate (Figure 4).

A metabolite-specific flip angle scheme can be used to adjust SNR for downstream metabolites in spsp acquisitions. For example, pyruvate flip angle is a trade-off between pyruvate and lactate SNR. Higher pyruvate flip angles result in more pyruvate signal, but the RF uses up more of the limited hyperpolarized signal and there is limited lactate signal. A smaller pyruvate flip angle scheme will result in higher lactate SNR (Figure 4 and Figure 5).

Spatial resolution can also be adjusted easily with a fast readout such as EPI. A finer resolution, although more advantageous for anatomical specificity, can result in lower SNR, although this is dependent on the metabolism within the animal, tumor, or organ imaged. Considerations for a coarser resolution may be appropriate for anatomy with lower perfusion, such as bone, and when vasculature is further from the ROI.

Other parameters may also need to be considered for spspEPI studies that were not explored in this work. For example, the imaging delay after start of injection was kept constant at 10 s. Shorter delays may capture more of the metabolic signal and lead to better quantification and pharmacokinetic model fitting.

### 4.4. Pharmacokinetic Modelling and Quantification

One method of quantification for hyperpolarized ^13^C-Pyruvate imaging is to use a pharmacokinetic model to fit for the apparent rate constant, *k_PL_*. This biomarker holds promise to be comparable across different acquisition methods and timings. In this study, the generality of *k_PL_* was demonstrated as it remained consistent across different acquisitions (CSI vs. EPI), different days of acquisition, and different mice for the same tumor type (Figure 6). The values were also consistent to previous studies using the same pharmacokinetic model for in vivo RCC tumors [27].

## 5. Conclusions

In this study, a 2D metabolite-specific EPI sequence was developed, calibrated, and tested for use on a preclinical Bruker system. A spectral–spatial pulse for hyperpolarized [1-^13^C]pyruvate was tested and implemented for acquisition. PSF simulations and in vivo data suggest this developed spspEPI sequence may be more favorable for preclinical studies in comparison to CSI sequences, due to minimized blurring from signal loss across phase encoding. The spspEPI sequence parameters (flip angles, temporal, and spatial resolution) were optimized to improve SNR and rate constant fitting precision using simulations and found to provide good results with in vivo experiments. Pyruvate to lactate rate constant, *k_PL_*, fitting using a pharmacokinetic model revealed comparable values across sequences, days, mice with similar tumors, and the previous literature [27].

## Data Availability

Links to pulse sequences, processing scripts, representative data, and codes (for data processing and simulation) can be found on https://coclinicalimaging.ucsf.edu accessed on 18 March 2023. The data presented in this paper will be made available upon reasonable request to the Corresponding Authors.

## Supplementary Materials

The following supporting information can be downloaded at: https://www.mdpi.com/article/10.3390/tomography9020059/s1, Figure S1: Average pyruvate and lactate CSI dynamics across tumor for four different mice. For all of these acquisitions, a CSI flip angle of 10 degrees was used.
