# Supplementary material for: Metabolite-Specific Echo Planar Imaging for Preclinical Studies with Hyperpolarized 13C-Pyruvate MRI
Source: Tomography. 2023 Mar 27;9(2):736–49. doi: 10.3390/tomography9020059 (PMC10143874; doi:10.3390/tomography9020059)
Supplement: Supplementary file 1 [file tomography-09-00059-s001.zip › tomography-2214207-supplementary.pdf]

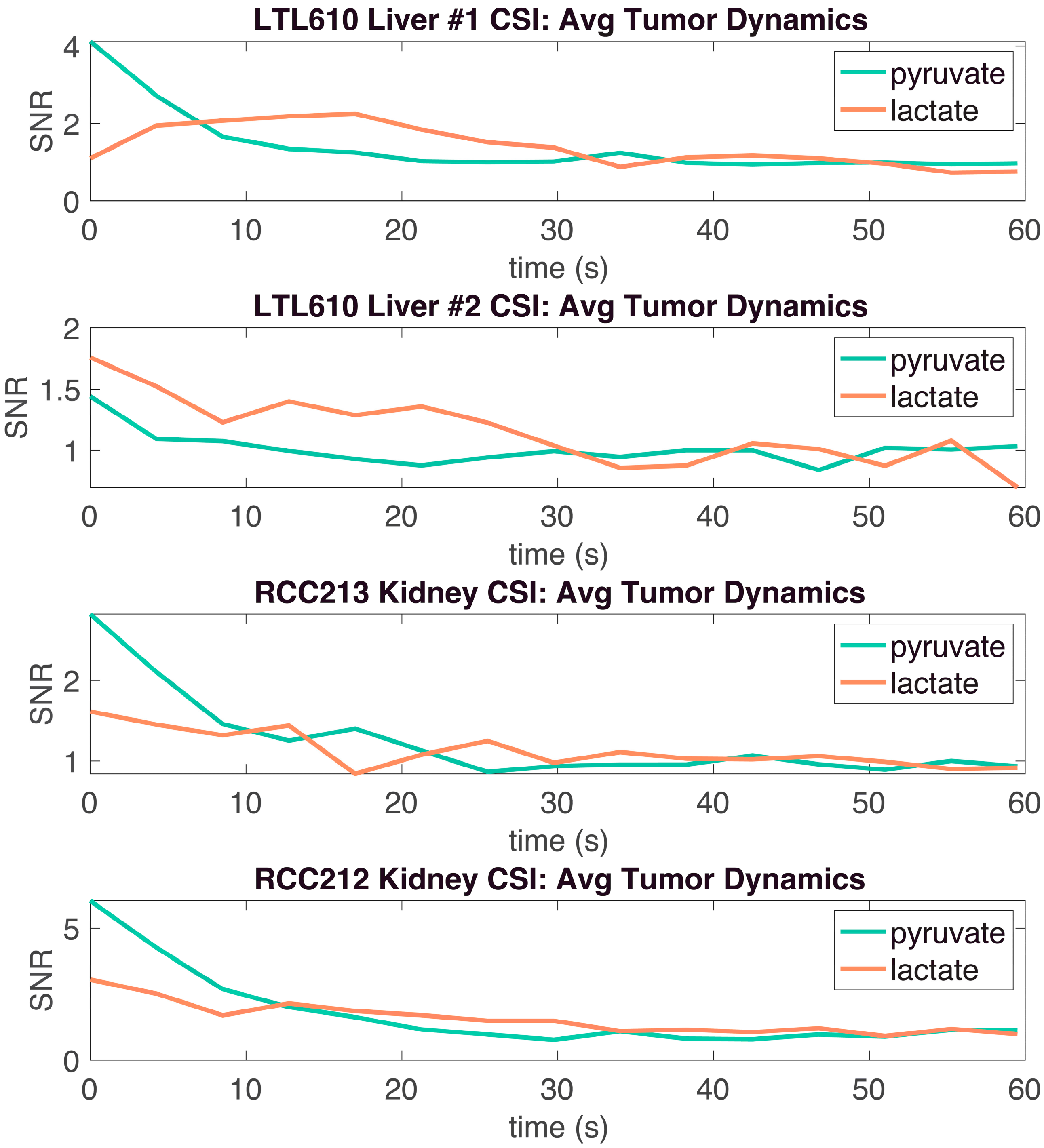

Figure S1. Average pyruvate and lactate CSI dynamics across tumor for four different mice. For all of these acquisitions a CSI flip angle of 10 degrees was used.
